# Yttrium-90 transarterial radioembolization for liver metastases from medullary thyroid cancer

**DOI:** 10.1530/ETJ-22-0130

**Published:** 2022-09-20

**Authors:** Luciana Puleo, Laura Agate, Irene Bargellini, Giuseppe Boni, Paolo Piaggi, Claudio Traino, Tommaso Depalo, Giulia Lorenzoni, Francesca Bianchi, Duccio Volterrani, Sandra Brogioni, Valeria Bottici, Maurizia Rossana Brunetto, Barbara Coco, Eleonora Molinaro, Rossella Elisei

**Affiliations:** 1 Endocrine Unit, Department of Clinical and Experimental Medicine; 2 Department of Vascular and Interventional Radiology; 3 Regional Center of Nuclear Medicine; 4 Hepatology Unit, University of Pisa, Pisa, Italy

**Keywords:** yttrium-90, TARE, medullary thyroid cancer, liver metastases, SIRT

## Abstract

**Objectives:**

Liver metastases occur in 45% of patients with advanced metastatic medullary thyroid cancer (MTC). Transarterial radioembolization (TARE) has been proposed to treat liver metastases (LM), especially in neuroendocrine tumors. The aim of this study was to investigate the biochemical (calcitonin and carcino-embryonic antigen) and objective response of liver metastases from MTC to TARE.

**Methods:**

TARE is an internal radiotherapy in which microspheres loaded with β-emitting yttrium-90 (^90^Y) are delivered into the hepatic arteries that supply blood to LM. Eight patients with progressive multiple LM underwent TARE and were followed prospectively. They were clinically, biochemically and radiologically evaluated at 1, 4, 12 and 18 months after TARE.

**Results:**

Two patients were excluded from the analysis due to severe liver injury and death due to extrahepatic disease progression, respectively. One month after TARE, a statistically significant (*P* = 0.02) reduction of calcitonin was observed in all patients and remained clinically relevant during follow-up; reduction of CEA, although not significant, was found in all patients. Significant reduction of liver tumor mass was observed 1, 4 and 12 months after TARE (*P* = 0.007, *P* = 0.004, *P* = 0.002, respectively). After 1 month, three of six patients showed partial response (PR) and three of six stable disease (SD) according to RECIST 1.1, while five of six patients had a PR and one of six a SD according to mRECIST. The clinical response remained relevant 18 months after TARE. Excluding one patient, all others showed only a slight and transient increase in liver enzymes.

**Conclusions:**

TARE is effective in LM treatment of MTC. The absence of severe complications and the good tolerability make TARE a valid therapeutic strategy when liver LM are multiple and progressive.

## Introduction

Medullary thyroid cancer (MTC) is a neuroendocrine tumor arising from parafollicular or calcitonin-producing C cells that retain the biochemical and pathological features of the cells from which it derives. MTC accounts for 3–5% of all thyroid cancer and can occur either in a sporadic (about 75% of cases) or in a hereditary form (about 25% of cases) (1). The biological behavior of MTC is less favorable than that of differentiated thyroid cancer, but a better prognosis can be achieved in those MTC with an early diagnosis and treatment (1, 2, 3). Nevertheless, about 10% of patients have distant metastases at diagnosis and this is the subgroup with the worst prognosis (4). According to the American Thyroid Association (ATA) guidelines, after surgery, serum calcitonin (Ct) and carcino-embryonic antigen (CEA) must be periodically tested in order to assess disease status, particularly when serum Ct is >150 pg/mL (2). MTC metastasizes most commonly to the liver, lungs, bone and more rarely to the skin and brain. Liver metastases occur in 45% of patients with advanced metastatic MTC, and when increasing either in number and/or in size, treatment is indicated (5). When surgical resection of liver metastases is not feasible, other local therapies can be considered such as percutaneous ethanol ablation, external radiation therapy or radiofrequency ablation (2). However, since liver metastases are often multiple and disseminated, transarterial treatments (such as embolization or chemoembolization) and systemic therapy can be the only therapeutic options (2).

In last decades, transarterial radioembolization (TARE), also known as selective internal radiation therapy (SIRT) (6, 7), with microspheres labeled with the β-emitting radioisotope yttrium-90 has been used for the treatment of inoperable liver malignancies (8, 9). In the last decade, this technique has been proposed mostly for the treatment of primary liver carcinomas (10, 11, 12) and liver metastases from colorectal cancer (10, 13, 14). The aim of TARE is a selective internal radiotherapy in which high-energy β-particles emitted by ^90^Y determine radiation-induced tumor necrosis. This technique involves the administration of glass or resin ^90^Y-labeled microspheres through a microcatheter selectively positioned into the artery(s) supplying the tumor lesions to be treated (9, 15, 16). Some authors reported the use of TARE for the treatment of unresectable liver metastases derived from neuroendocrine tumors (NETs), suggesting it as a safe and efficient treatment procedure in this specific context (6, 17, 18, 19).

On the basis of this evidence and in consideration of the high vascularization of liver metastases from MTC, as well as its neuroendocrine origin, a group of patients with unresectable liver metastases was selected for TARE. The aim of this study was to evaluate the efficacy and the safety profile of TARE in patients with liver metastases from MTC by investigating the biochemical (serum Ct and CEA) and objective response of tumoral lesions.

## Patients and methods

### Patients

The study was conducted at the Unit of Endocrinology in collaboration with the Unit of Nuclear Medicine and the vascular Interventional Radiology of the University Hospital of Pisa. Eight patients, six males and two females, at a mean age of 51 years (range: 31–73 years) with liver metastases from MTC were included in the study. The decision to perform the TARE treatment was taken by a dedicated multidisciplinary team based on the evidence of the progression (both numerical and in size) of liver metastases. The TARE procedure was performed in collaboration with nuclear medicine physicians and interventional radiologists at the University Hospital of Pisa. Inclusion criteria: (i) sporadic advanced metastatic MTC with hypervascular progressive and/or symptomatic multiple liver metastases (with less than 50% of hepatic involvement); (ii) normal liver function and (iii) liver-only or liver-dominant metastatic disease. All patients underwent TARE between April 2017 and May 2018. Two patients were treated with TKI (vandetanib) before TARE, whereas six patients were naïve to any anticancer treatment. No TKI treatment was simultaneously performed during TARE procedure. Data were prospectively collected and analyzed at the end of the study.

All patients gave their signed approval for the use of their personal data for research and scientific purposes as part of the policy of our University Hospital. Moreover, patients signed a specific consent to perform the angiographic and TARE procedure and the study was approved by the local Ethics Committee (Comitato Etico Area Vasta Nord Ovest, protocol number 24059). The study was conducted in accordance with the provisions of the Declaration of Helsinki (2013).

### Patient’s pretreatment evaluations

Before treatment, all patients underwent baseline screening with a metabolic profile including liver function tests (total bilirubin, alanine aminotransferase (ALT), aspartate aminotransferase (AST), gamma-glutamyltransferase (GGT), alkaline phosphatase (ALP), albumin and international normalized ratio (INR)) and complete blood count.

The measurement of Ct (IMMULITE 2000 System Analyzers, Siemens Healthcare Diagnostics Products; normal range: males until 18.2 pg/mL; females until 11.5 pg/mL) and CEA (ELECSYS CEA, Roche Diagnostics; normal range: <5.2 ng/mL) was performed at baseline and at any subsequent evaluation.

Patients underwent either CT or MRI to evaluate the total volume of the liver and hepatic tumor volume (HTV). The estimated HTV was calculated using a dedicated semi-automated CT software (WorkStation ADW 4.7—GE Healthcare) that contours lesions for quantitative analysis. The Interventional Radiologist selected each lesion in the liver and the software calculated the total tumor volume in cm^3^. In order to evaluate the feasibility of TARE, angiography was performed a few days before the procedure. Technetium 99m-labeled macroaggregates of albumin (^99m^Tc-MAA) (Technescan®, LyoMAA, Mallinckrodt Medical, Petten, the Netherlands) were injected into the selected hepatic arteries or branches during the angiography procedure to evaluate the intrahepatic activity distribution, to calculate lung shunt fraction (LSF) (20, 21) and to exclude the presence of abdominal extrahepatic shunts (aberrant vasculature to the liver and gastrointestinal tract). Whole-body acquisitions and SPECT/CT scans of the upper abdomen were performed with a Discovery NM/CT 670 (GE Healthcare) scanner within 1 h of the ^99m^Tc MAA injection. Only patients without any collateral arterial flow to the gastrointestinal tract and with an LSF <20% were treated. Both angiography and TARE, as reported in the following paragraph, were performed by the Interventional Radiologists (BI, GL) in collaboration with nuclear medicine doctors (GB, FB, TD).

### Transarterial radioembolization procedure

TARE was performed within 14 days from the diagnostic angiography. The administered activity was determined according to the manufacturers’ recommendations, using standard body surface area (BSA) method. ^90^Y resin microspheres (SIR-Spheres®, Sirtex Medical Products, Sydney, Australia) were then injected during the angiographic procedure into the hepatic artery of interest, placing the microcatheter in the same position as the ^99m^Tc-MAA injection. Finally, patients were discharged the day after the procedure. In the presence of bilobar extension of liver metastases, patient carried out two subsequent lobar procedures at an interval of approximately 1 month. Within 24 h after each treatment, a PET/CT acquisition of the upper abdomen was performed (Discovery 710; GE Healthcare Milwaukee) to verify the actual distribution of microspheres within the liver. The freeware software ImageJ (Wayne Rasband, National Institute of Health, USA) was used to process transaxial PET scans of each treatment in order to calculate the average absorbed dose delivered to the tumor and healthy targeted liver by voxel-based dosimetry. The normalized average absorbed dose method (NAAD) processed the counting density in selected volumes of interest (VOI; for example, tumor or healthy targeted liver) and then, the average absorbed dose was calculated by the classic medical internal radiation dose (MIRD) formula, reported below:


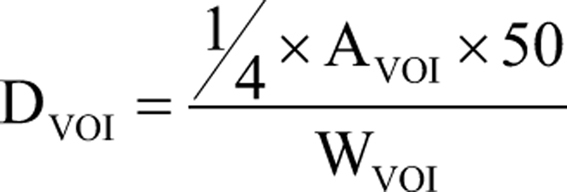


where D_VOI_ was the average dose into the VOI expressed in gray (Gy); A_VOI_ was the total activity into the VOI expressed in giga-becquerel (GBq); W_VOI_ was the weight of the VOI expressed in kg and obtained from the product between the volume of VOI and the tissue density (about 1.03 g/mL). For this procedure, we have considered the institutional radiation safety guidelines.

Average tumor absorbed dose calculation was feasible in seven of eight patients since, in one patient, the acquisition of PET/CT was not performed for technical reasons.

### Post-treatment evaluations

All patients were followed through a pre-established scheduled program. Clinical evaluation, lab tests (liver function, serum Ct and CEA measurements) and imaging (CT scan or MRI) were performed 1, 4, 12 and 18 months after the TARE procedure.

Objective response to TARE was based on both the Response Evaluation Criteria in Solid Tumors version 1.1 (RECIST 1.1) (22, 23) and ‘modified’ Response Evaluation Criteria in Solid Tumors (mRECIST) (24). Both criteria were used since RECIST 1.1 evaluate the increase or reduction in size of target lesion(s), while mRECIST evaluate the tumor necrosis and the devascularization of hypervascularized metastases such as those of the neuroendocrine tumors, including MTC that is sometimes more significant than the size reduction (25). This evaluation was performed by two independent radiologists (BI, GL).

The effects of TARE on liver function were also evaluated by expert hepatologists (BM and CB) by performing liver ultrasound and measuring total bilirubin, ALT, AST, GGT and ALP. Moreover, the grading of hepatotoxicity was assessed according to the National Cancer Institute Common Terminology Criteria for Adverse Events (CTCAE Version 4.0) (26).

### Statistical analysis

Statistical data analysis was performed using the software SPSS (version 21; IBM Corp.). The Shapiro–Wilk test was used to assess normality of data distribution. When possible, skewed variables were log-transformed to approximate a normal distribution. The changes in each parameter from baseline at each follow-up visit were evaluated by paired *t*-test or Wilcoxon test for Gaussian and skewed variables, respectively. The χ^2^ test or the Fisher exact test was used to assess differences in counts and frequency when appropriate. Alpha level was set at 0.05. Data are presented as mean ± s.d. (Gaussian variables), geometric mean with 95% CI (log-transformed variables) or frequency (percentage).

## Results

As shown in Table 1, four of eight (50%) patients received one treatment (right lobe), one of whom underwent TARE treatment of the right lobe followed by bland embolization of a single lesion in the left lobe; three of eight (37.5%) patients received bilobar treatment in two different sessions, while in one patient (12.5%) a whole-liver TARE was performed. The mean total hepatic tumor volume was 103 ± 183 (SD) mL (median 31.5 mL; range: 12–551 mL). In seven patients, the mean tumor absorbed dose was 235 ± 205 (SD) Gy (median 144 Gy; range: 90–677 Gy). Six out of eight patients have a complete follow-up of 18 months after TARE. Two patients were excluded from the analysis because of too short follow-up in one case (> 2 months) who died due to the severity of the disease mainly involving an extrahepatic disease progression and, in the other, for the onset of a rather severe liver insult that did not allow the patient to continue the follow-up.

**Table 1:** Clinical-pathological features and treatment of the MTC study group.

Sex	
Male	6/8 (75%)
Female	2/8 (25%)
Age, mean	51 ± 14.5 years (range: 31–73)
Medullary thyroid cancer	
Sporadic	8/8 (100%)
Hereditary	0
Extrahepatic metastases	
Laterocervical and mediastinal lymphnodes	7/8 (87.5%)
Lung	3/8 (37.5%)
Bone	1/8 (12.5%)
Total hepatic tumor volume, mean	103 ± 183 mL (range: 12–551)
Administered dose, mean	235 ± 205 Gy (range: 90–677)
Treated liver lobe	
Only right lobe^1^	4/8 (50%)
Bilobar (right and left lobe in two times)	3/8 (37.5%)
Bilobar (right and left lobe in one time)	1/8 (12.5%)
Previous treatment	
Tyrosine kinase inhibitors	2/8 (25%)

^1^In one of these patients, embolization (TAE) was performed at the level of the left lobe of the liver.

**Figure 1:**
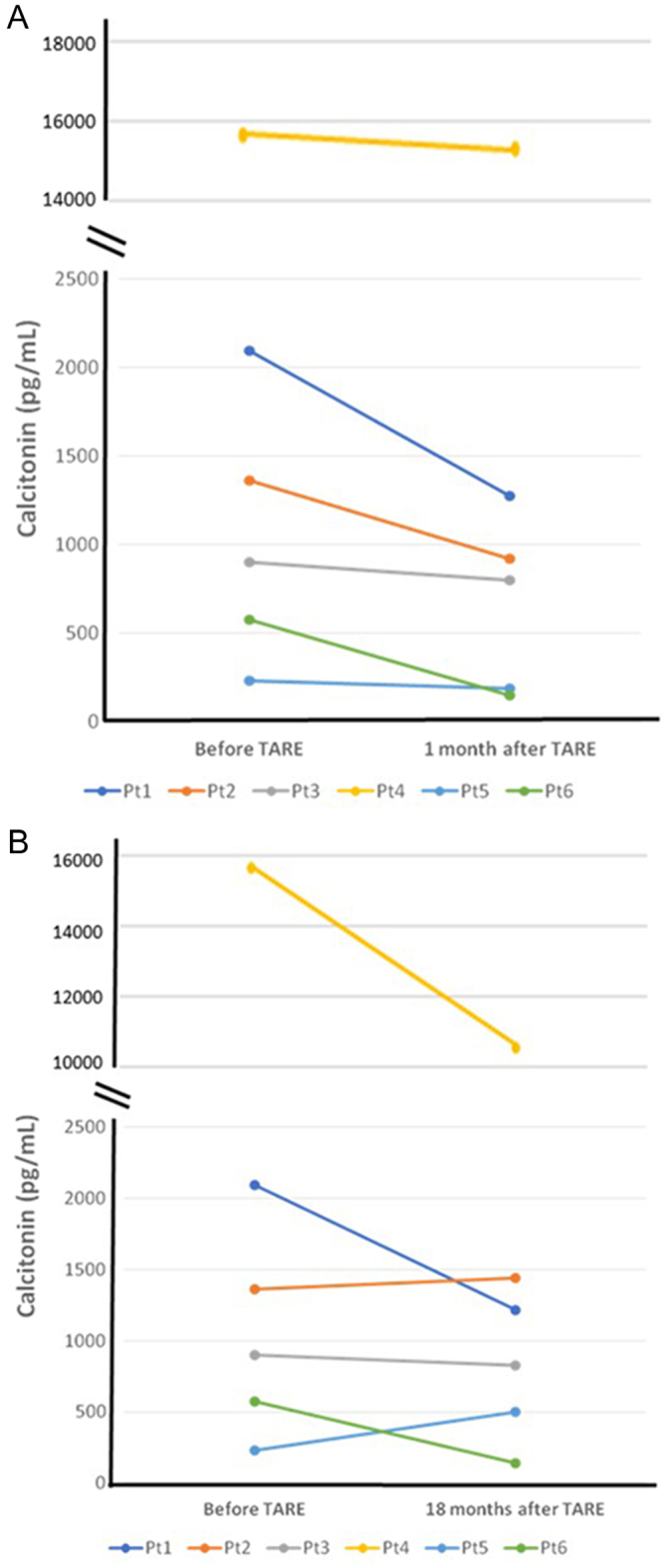
Trend of serum Ct before, 1 month (A) and 18 months (B) after TARE in six patients with an 18 months follow-up.

One month after TARE, a statistically significant reduction (*P* = 0.02) of the Ct values was observed in all patients (Fig. 1, panel A). At the end of follow-up (18 months), the reduction of Ct was still relevant although not more statistically significant (Fig. 1, panel B).

**Figure 2:**
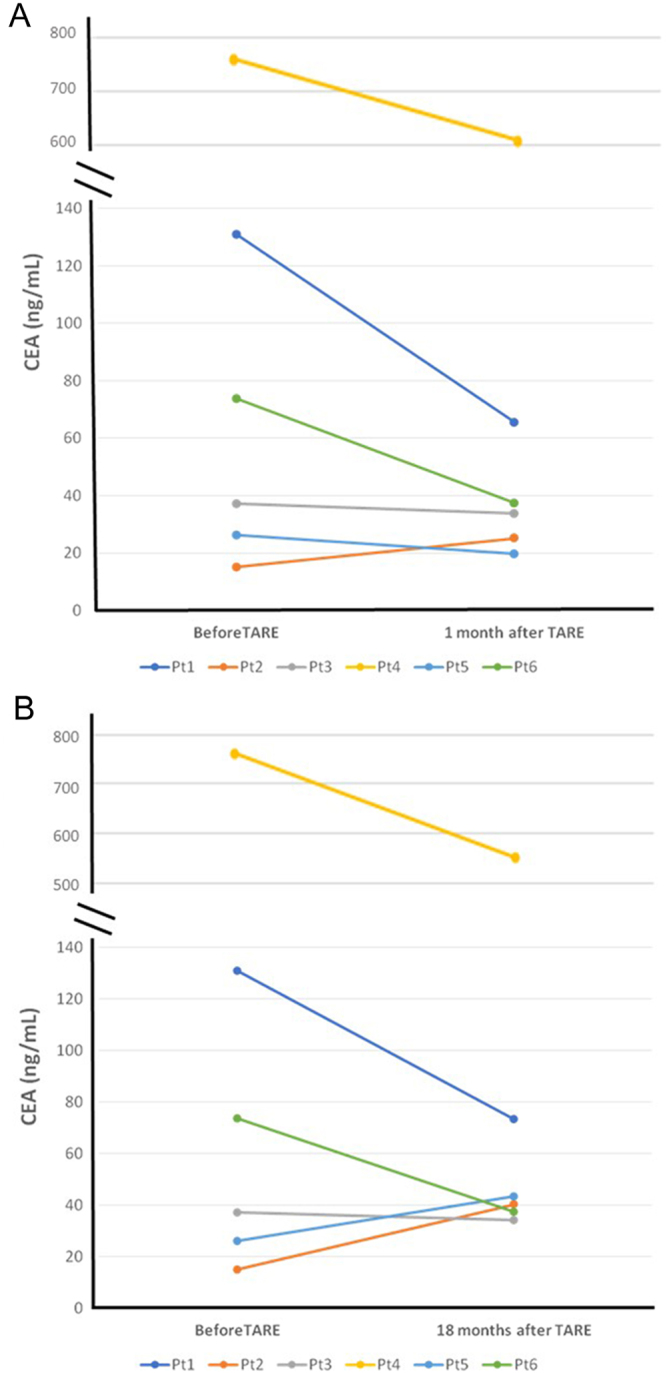
Trend of serum CEA before, 1 month (A) and 18 months (B) after TARE in six patients with an 18 months follow-up.

As shown in Fig. 2, we observed a trend of CEA reduction in all patients during the entire follow-up, although the difference was not statistically significant.

**Figure 3:**
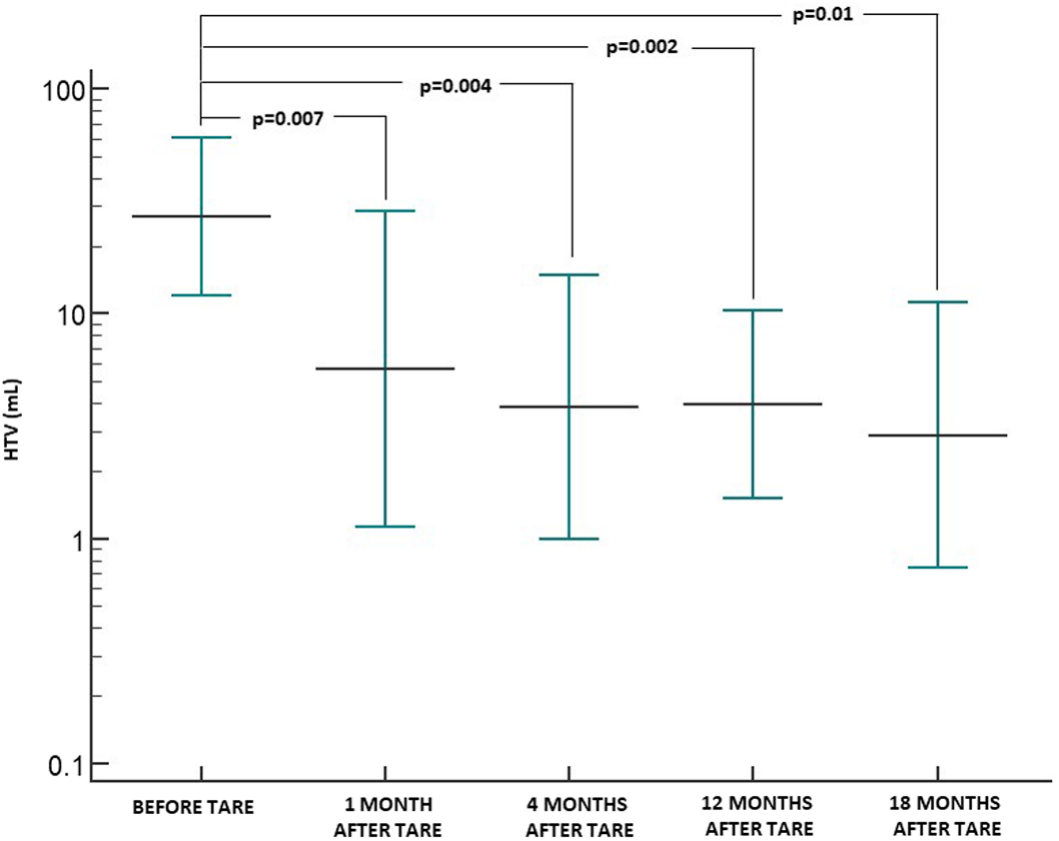
Trend of hepatic tumor volume (HTV) before and during the follow-up after TARE (error bars show geometric means with 95% CI).

The mean ± s.d. of pre-TARE total hepatic tumor volume of the six patients who completed the follow-up was 30.2 ± 19.5 mL (median: 23 mL; range: 12–66 mL). We observed a statistically significant reduction (*P* = 0.007) of the total hepatic tumor volume in all six patients 1 month after TARE that persisted after 4, 12 and 18 months (*P* = 0.004; *P* = 0.002 and *P* = 0.01, respectively) as shown in Fig. 3. Compared to baseline (geometric mean = 25.9 mL, 95% CI: 13.9–48.2 mL), the volume decreased on average by 77.2% after 1 month (geometric mean = 5.9 mL, 95% CI: 1.7–20.1 mL), by 84.6% after 4 months (geometric mean = 4.0 mL, 95% CI: 1.4–11.1 mL), by 83.8% after 12 months (geometric mean = 4.2 mL, 95% CI: 2–8.8 mL) and by 88% after 18 months (arithmetic mean = 3.75 mL, 95% CI: 1.2–8.7 mL).

**Figure 4:**
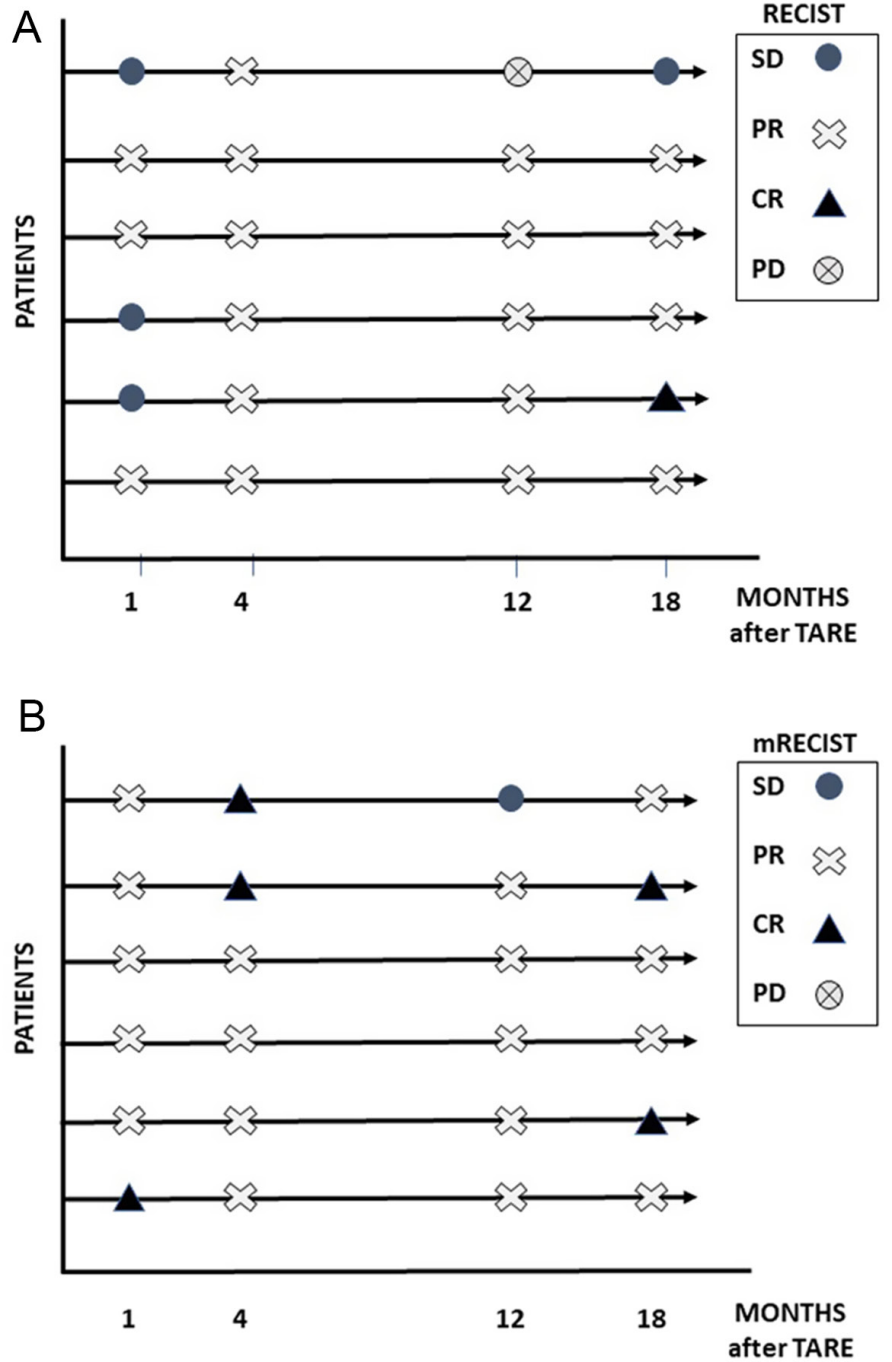
Response of liver metastases to TARE treatment according to RECIST 1.1 (A) and mRECIST (B).

**Figure 5:**
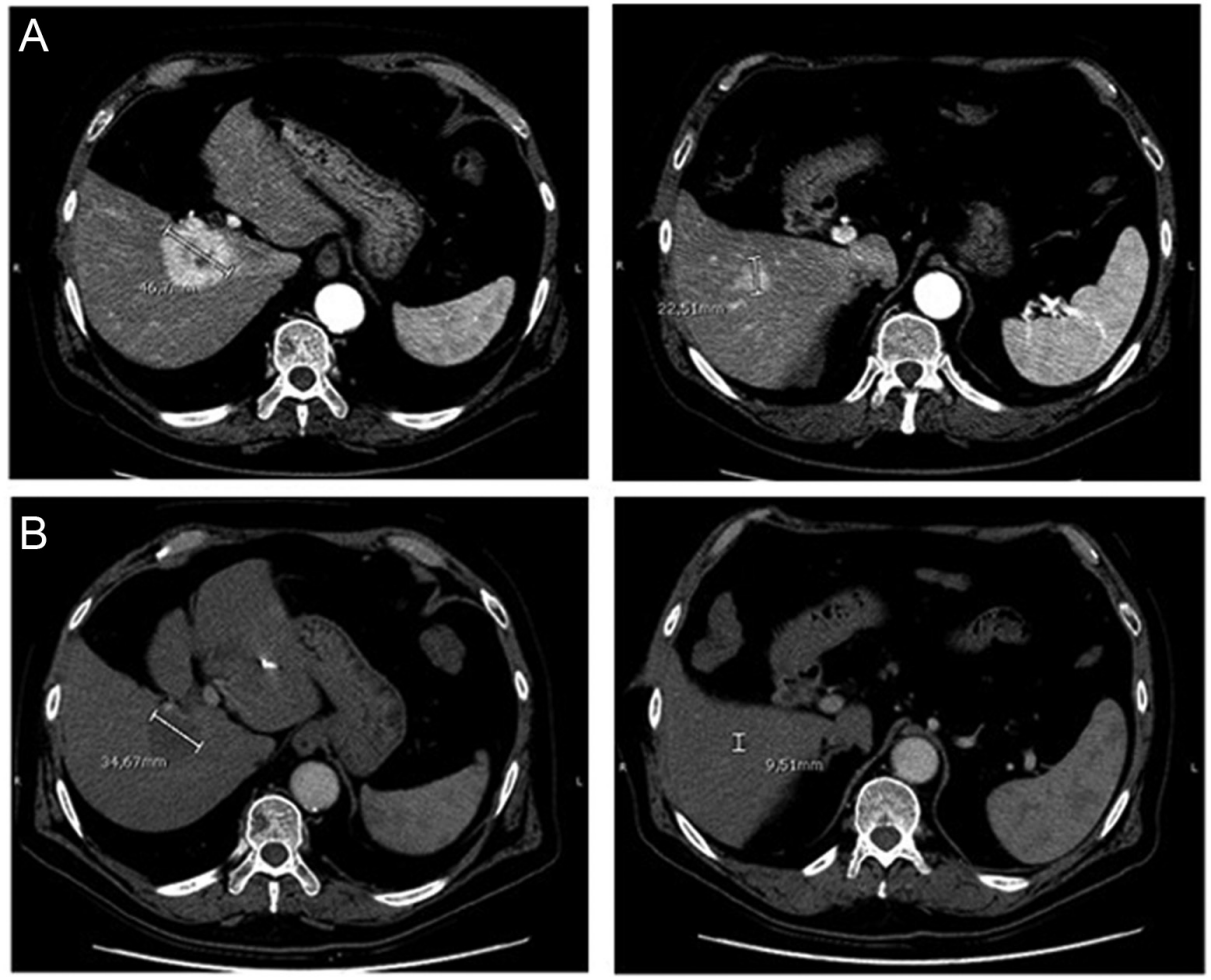
CT scan images of a patient with liver metastases of MTC in the right hepatic lobe before (A) and 1 month after TARE (B). CR was documented according to mRECIST.

According to the RECIST 1.1, partial response (PR) was observed in three of six patients and stable disease (SD) in the other three patients at the first evaluation (Fig. 4, panel A). After 18 months, one patient showed complete response (CR), four of six patients had a PR and one patient maintained the previous SD. According to mRECIST, 1 month after treatment five of six patients had PR and one of six patients showed CR, while at 18 months, a PR was observed in four of six patients and a CR in two of six cases (Fig. 4, panel B). The case with CR according to mRECIST who showed PR according to RECIST 1.1 is shown in Fig. 5. None of the six patients developed new lesions during the 18 months of follow-up and none of them required to start systemic therapy during this time.

As regards the hepatotoxicity of this treatment assessed by the criteria of CTCAE Version 4.0 (26), all eight enrolled patients had normal serum values of bilirubin, AST, ALT, GGT, ALP, albumin and INR before treatment. In all six patients who completed the follow-up, bilirubin, albumin and INR levels remained in the normal range after treatment and during the follow-up; no severe alterations in liver function were observed with only mild G1 and G2 variations of transaminases that were resolved within 12 months in the majority of cases (Table 2). Only one patient, the one treated with a whole-liver TARE, experienced an asymptomatic liver insult (i.e. moderate increase of AST, ALT and GGT associated to a mild hepatic hypotrophy) 2 months after TARE. Nevertheless, the clinical and biochemical resolution was reached approximately 5 months after TARE. At latest follow-up (2 years after TARE), the patient has SD at CT scan and a persistent slight increase (G1) of AST, ALT and GGT values. No other severe treatment-related adverse events were observed.

**Table 2:** Hepatotoxicity over time after TARE (CTACE version 4.0)^*^ in MTC patients (*n*  = 6).

Value	Grade	Baseline	1 month FU	4 months FU	12 months FU	18 months FU
Bilirubin	G0	6/6	6/6	6/6	6/6	6/6
Albumin	G0	6/6	6/6	6/6	6/6	6/6
AST (normal value up to 45 U/L)	G0	6/6	3/6	5/6	5/6	5/6
	G1	–	3/6	1/6	1/6	1/6
	G2	–	–	–	–	–
ALT (normal value up to 40 U/L)	G0	6/6	1/6	3/6	4/6	5/6
	G1	–	5/6	3/6	2/6	1/6
	G2	–	–	–	–	–
GGT (normal value up to 60 U/L)	G0	6/6	2/6	1/6	1/6	2/6
	G1	–	3/6	1/6	3/6	4/6
	G2	–	1/6	4/6	2/6	–
ALP (normal value 30–130 U/L)	G0	6/6	3/6	2/6	6/6	6/6
	G1	–	3/6	4/6	–	–
	G2	–	–	–	–	–
INR	G0	6/6	6/6	6/6	6/6	6/6

^*^CTACE, Common Terminology Criteria for Adverse Events (ref. (22)): G0 is arbitrarily referred to normal values.

ALP, alkaline phosphatase; ALT, alanine aminotransferase; AST, aspartate aminotransferase; GGT, gamma-glutamyltransferase; INR, international normalized ratio.

## Discussion

Liver metastases occur in 45% of patients with advanced metastatic MTC (5). When liver metastases are multiple and disseminated, surgery and percutaneous ablation techniques are not recommended. In this clinical context, and so far, the only therapeutic option could be chemoembolization or systemic therapy (2). At the present, there are two drugs, vandetanib (27) and cabozantinib (28), belonging to the group of tyrosine kinase inhibitors, that can be used for multimetastatic and progressive MTC. However, these drugs have several side effects that can highly impact the quality of life of these patients (29, 30). For this reason, there is an indication to apply local therapies before starting systemic therapy (5) whenever possible. In this regard, none of all six patients followed up for 18 months required to be treated with systemic therapy during this period, confirming that TARE treatment allowed them to gain months free from systemic therapies and their side effects.

In recent years, TARE has been proposed as a new technique of locoregional intra-arterial therapy for the treatment of liver metastases in several solid human tumors such as primary liver carcinomas and liver metastases from colorectal cancer and neuroendocrine tumors (11, 13, 17, 19).

To our knowledge, this is the first study showing the feasibility of this procedure for the treatment of liver metastases from MTC based on the rationale that MTC is a neuroendocrine tumor. Our study showed a statistically significant reduction of serum Ct values 1 month after TARE. This reduction persisted throughout the follow-up and at least until 18 months, although the statistical significance was lost. The same results were observed for the CEA values that showed a trend of reduction although not statistically significant. It is known that serum Ct and CEA values are very good markers of the disease and they correlate with the tumor burden and their changes can be used as predictors of good or bad response to treatment (31). In our cases, the biochemical response was in line with the radiological one. It is well known that ^90^Y-labeled microspheres remaining permanently implanted within the vasculature of tumoral lesions cause radiation-induced tumor necrosis, preserving the remaining liver parenchyma (32). This would be in line with our result; in fact, the reduction of Ct and CEA could be explained by the death of tumoral cells following therapy. The fact that Ct and CEA values decrease in a non-statistically significant way could be explained by the fact that all patients also have metastases in lymph nodes and some even in bones and lungs. As previously said, Ct and CEA levels are correlated with the tumoral burden and tumor growth rate (33) and we can not distinguish from which metastases they derive. It is conceivable that the reduction of both markers due to the liver metastases damage was at least in part masked by the Ct and CEA secretion due to the other metastatic lesions.

Regarding the structural response of liver metastases to TARE, our data showed the reduction of the total liver tumor volume in all patients already from the first month after TARE and up to 18 months of follow-up. The liver metastases response to TARE was even better evaluated when we considered mRECIST that showed four patients with CR with respect to only one when evaluated by RECIST1.1. It is important to consider that TARE determines characteristic perilesional alterations such as edema, ring enhancement and ill-defined areas of hypoattenuation which are to be considered as a response, but which could induce an underestimation of the real response. In fact, after TARE, the tumor may shrink, remain the same or even increase in size for the necrosis, edema and hemorrhage that may persist for several months (34). This phenomenon explains the difference in the results obtained by either RECIST1.1 or mRECIST. In our opinion, since liver metastases from MTC are highly vascularized and considering the neuroendocrine nature of the same, it would be more appropriate to use the mRECIST for the evaluation of the response to this therapy (24).

Liver cell damage from treatment radiation can be a severe complication of this treatment. In one case, we had a rather severe and acute liver insult that severely affected the patient although it was fully resolved 3 months later. This patient was the only one in whom both lobes were treated at one single time. It is evident that the radiation total activity delivered to the entire liver in one single shoot was too toxic. In this regard, it is important underlying that in the other patients, we observed only a slight increase in liver enzymes that is indicative of low-mild liver cell damage from treatment-induced radiation. This data confirm the low hepatotoxicity of TARE if performed at least in two times (6, 35).

This study presents two major limitations due to the small number of the group of patients and the absence of long-term follow-up. The small number of patients is related to the rarity of MTC and in particular, of advanced metastatic MTC; moreover, we included only patients with a significant number of liver metastases and progressing. Nevertheless, in our study, liver metastases from MTC respond well to treatment with TARE, within a reasonable safety profile and the study can be considered a pilot study to be improved in the next future. As far as the length of follow-up is concerned, we are conscious that 18 months are not so many but it is still relevant that, in this period of time, none of our patients enter into a further progression despite their advanced disease.

In conclusion, our study suggests that TARE might be a safe and effective treatment for liver metastases from MTC, as it happens in other neuroendocrine tumors. Although we know that our results need to be validated in a larger cohort of MTC patients, this study shows that the TARE treatment of liver metastases from MTC allows to postpone the use of systemic therapies, such as tyrosine kinase inhibitors, that are known to have several side effects impairing the quality of life of these patients.

## Declaration of interest

B I is consultant for Sirtex Medical, Boston Scientific, BTG Biocompatible Ltd, Terumo. The other authors have nothing to disclose.

## Funding

This work has been supported by Associazione Italiana Ricerca sul Cancro (AIRC, Investigator grant 2018, project code 21790).

## Author contribution statement

Conceptualization, L P, L A, I B, C T and R E; Data curation, L P, L A, D T and G L; Formal analysis, L P, L A, G B, M B, B C; Investigation, L P, L A, S B; Methodology, P P, I B, G L, G B, F B; Supervision, R E; Writing – original draft, L P and L A; Writing – review and editing, L P, L A and R E. Visualization: S B, V B, D V and E M. All authors have read and agreed to the published version of the manuscript.
